# Timing of Nephrology Referral: Influence on Mortality and Morbidity in Chronic Kidney Disease

**DOI:** 10.5812/numonthly.2232

**Published:** 2012-06-20

**Authors:** Sushanth Kumar, Jayakumar Jeganathan, Amruthesh

**Affiliations:** 1 Department of Medicine, Kasturba Medical College- Mangalore, Manipal University, Mangalore, India

**Keywords:** Renal Insufficiency, Nephrology, Mortality, Morbidity

## Abstract

**Background:**

Few studies in India as well as in most developing countries have compared the mortality and morbidity rates between chronic kidney disease patients who were referred early to nephrologists and those who were referred late.

**Objectives:**

To study the mortality and morbidity patterns and to compare the various clinical parameters between the abovementioned early and late referrals.

**Patients and Methods:**

Fifty consecutive chronic kidney disease patients were followed up for one year. They were then classified as early referral (patients who underwent dialysis more than three months after the referral) and late referral (patients who underwent dialysis within three months of the referral). Clinical, laboratory parameters, and mortality patterns were compared between the two groups.

**Results:**

The blood pressure, hemoglobin, glomerular filtration rate, and calcium and phosphate values were better in the early referral group. Among the 24 complications that occurred, 17 (70.8%) were seen among the patients who were referred late. Among the 13 deaths that occurred, only one belonged to the early referral group.

**Conclusions:**

We observed that the mortality rate and clinical parameters were better in patients who were referred early to nephrologists.

## 1. Background

Chronic kidney disease is a growing problem worldwide (1). The timing of nephrology referral in the predialysis stage of chronic kidney disease is increasingly being considered as an important variable related to prognosis (2). The definition of “late” is somewhat arbitrary and varies in the literature, ranging from less than one month to one year before kidney replacement (3). One argument for early evaluation is that the management of chronic renal insufficiency and its complications may be improved (4). Early evaluation might lead to improved patient education about dialysis, provide more time for an informed decision about the type of dialysis, and permit timely placement of permanent vascular access (4). However, with the growing number of chronic kidney disease patients and the limited number of nephrologists available in most developing countries including India (5), we need to determine whether the predialysis stage can be managed effectively by the primary physicians. Unfortunately, there is a paucity of data in India and in most developing countries regarding the difference in the outcome of these patients based on the timing of the nephrology referral (6). This study is intended as a small step in that direction.

## 2. Objectives

1. To study the morbidity and mortality patterns in chronic kidney disease patients referred early vs. late to nephrologists.

2. To compare the various clinical parameters between the early and late referral groups.

## 3. Patients and Methods

We conducted a prospective study in our tertiary care hospital in southwest India. Fifty chronic kidney disease patients (definition described below) were selected based on our inclusion and exclusion criteria, and all of them were followed up for a period of one year. The method followed for the estimation of glomerular filtration rate (GFR) was that given by Modification of Diet in the Renal Disease Study Group (7). During the first visit, history about the present illness and past history, duration of renal disease, reasons for referral, detailed clinical examination, and relevant laboratory investigation (e.g., serum creatinine, hemoglobin, calcium, phosphate, albumin, and urine analysis) were determined. The same laboratory measurements were repeated during the follow up visits. The need for initial emergent dialysis, the mode of vascular access, and the duration between the first visit and initiation of dialysis were also noted. Patients were followed up, and all patients were re-examined at the end of the third month, sixth month, and twelfth month. The patients were divided into early and late referral groups as per the definition adopted in our previous study, and the clinical and relevant laboratory parameters assessed and the mortality pattern at these time points were compared between the two groups of patients.

### 3.1. Inclusion Criteria

1) Cases of chronic kidney disease referred to the nephrology department of our hospital; 2) Age more than eighteen years.

### 3.2. Exclusion Criteria

1) Patients with even a single visit to a nephrologist outside our hospital; 2) Patients already receiving treatment from a nephrologist in our hospital; 3) Patients who consulted the nephrologists in our hospital without any referral; 4) Chronic kidney disease patients with any underlying malignancy; 5) All cases of acute renal failure with or without underlying chronic kidney disease; 6) Chronic kidney disease patients who had already received dialysis outside our hospital and were then referred to our hospital.

### 3.3. Definitions Adopted in Our Study

1) Chronic kidney disease: Defined as GFR ≤ 60 mL/min/1.73 m^2^ for ≥ three months (8); 2) Definition of late and early referral: Chronic kidney disease patients who required the initiation of dialysis within three months of referral to the nephrologist were grouped as late referrals and those who required dialysis more than three months after the referral to the nephrologist were called early referrals (9); 3) Initial emergent dialysis: Defined as the need for dialysis within 24 hours of the referral.

### 3.4. Statistics

Clinical and laboratory parameters were compared between the early and the late referral groups using Student’s unpaired t-test. P ≤ 0.05 was considered significant. The mortality rates and referral pattern were also compared.

## 4. Results

Among the 50 cases, 35 patients (70%) were males and 15 (30%) were females. As shown in Table 1, systolic and diastolic blood pressure were both higher in the late referral group, but this difference was not statistically significant. Haemoglobin, GFR, and serum calcium were lower in the late referrals and the differences were statistically significant. Serum phosphate was also significantly increased in the late referral group. It was found that 53.1% (17/32) of the patients underwent initial emergent dialysis in the late referral group. Also, the mean duration between the time of referral and initiation of dialysis was 237 days in the early referral group and 33.6 days in the late referral group. Complications seen in the early and late referrals included cardiac (coronary artery disease, pulmonary edema, and pericarditis), respiratory (pneumonia and pleurisy), central nervous system (seizures, stroke, encephalitis, and peripheral neuropathy) and urinary afflictions and are shown in Tables 2, 3, 4. In the first three months of follow up, there were 10 complications, and all of them in the late referral group. In the next three months (3–6 months), there were eight complications, among which six (75%) occurred in the late referral group. In the last six months (6–12 months), there were six complications, among which five (83.3%) occurred in the early referral group. Overall, there were 24 complications, and 17 (70.8%) of them occurred in the late referral group. Among the 13 deaths that occurred during the one-year follow up, only one belonged to the early referral group.

**Table 1:** BaseLine Characteristics of the Early and Late Referral Group of Patients

	Early Referral, Mean ± SD (n = 18)	Late Referral, Mean ± SD (n = 32)	P value
Mean age, y	53.9 ± 5.6	60.9 ± 7.3	0.06
Systolic BP, mm of Hg	160 ± 17.8	162.8 ± 25.8	0.69
Diastolic BP, mm of Hg	90.4 ± 11.2	94.9 ± 10.1	0.15
Haemoglobin, g/dL	10.9 ± 1.03	9.4 ± 1.4	0.01 a
Mean GFR, mL/min	45.6 ± 5.8	11.4 ± 5.7	0.001 a
Calcium, mg/dL	8.5 ± 0.5	8 ± 0.9	0.02 a
Phosphate, mg/dL	4.4 ± 1.4	6.2 ± 2.7	0.01 a
Albumin, mg/dL	3.3 ± 0.4	3.2 ± 0.3	0.1

^a^ P value <0.05

**Table 2:** The Complications in the Early and Late Referral Groups at 3 Months

	Early Group No. (%)	Late Group No. (%)	Total No.
Cardiac	0 (0)	3 (100)	3
Respiratory	0 (0)	3 (100)	3
Neurological	0 (0)	2 (100)	2
Urinary	0 (0)	2 (100)	2

**Table 3:** Complications in the Early and Late Referral Groups at 3-6 Months

Complication	Early Group No. (%)	Late Group No. (%)	Total No.
Cardiac	1 (33.3)	2 (66.7)	3
Respiratory	0 (0)	2 (100)	2
Neurological	1 (50)	1 (50)	2
Urinary	0 (0)	1 (100)	1

**Table 4:** The Complications in the Early and Late Referral Groups at 6-12 Months

Complications	Early Group No. (%)	Late Group No. (%)	Total No.
Cardiac	2 (100)	0 (0)	2
Respiratory	0 (0)	0 (0)	0
Neurological	3 (75)	1 (25)	4
Urinary	0 (0)	0 (0)	0

## 5. Discussion

We studied fifty consecutive cases of chronic kidney disease and followed them for one year. The blood pressure, hemoglobin, calcium, phosphate, and albumin values were better in the early than in the late referral group. The differences in hemoglobin, calcium, and phosphate values were statistically significant. A meta-analysis by Chan et al. in 2007 also found that early referrals have low-level anemia and higher serum albumin (2). Both these factors were associated with better survival in end-stage renal disease subjects in that study. In our study, initial emergent dialysis was required for 53.1% of the late referrals. None of the early referrals required initial emergent dialysis. This may be an important factor determining the prognosis of these patients as shown by Abderrahim et al. for Tunisian patients (10), where the urgent initiation of dialysis was an independent risk factor for death. Frimat et al. (11) found that the commencement of dialysis in an emergency was associated with late referral. Fujimaki et al. also showed an increased need for urgent initial dialysis in late referrals (12). In our study, the maximum number of complications occurred during the first three months of follow up. Overall, in the whole year of follow up, 17 out of the 24 complications occurred in the late referral group. Jungers et al. in 2006 found that complications such as major cardiovascular events were twice as frequent in patients referred late to nephrologists (13). Ratcliffe et al. had reported that serious complications prolonging the length of hospital stay were significantly more frequent in the late referred group (14). At the end of the one-year follow up in our study, most of the deaths occurred in the late referral group. Chan et al., in a meta-analysis of 12,749 patients, found that late referral was associated with increased overall mortality (2). However, Ellis et al. found no significant difference in the mortality after one year between the early and late referral groups (15). Similarly, Roubicek et al. also showed that the survival at three months, twelve months, and five years was the same for both early and late referral groups (16). Thus, although most studies favored early referral to nephrologists, some did not find any significant advantage in doing the same. One of the reasons for this variation may be the geographic location, as the type of predialysis management by primary care physicians may vary from place to place and country to country. Hence, before formulating guidelines regarding the timing of referral to nephrologists, an evaluation of the results of studies performed in that country and location may be important.

In our study, we found that late referral gives rise to increased complications and mortality. So should all chronic kidney disease patients be referred early to nephrologists? The answer may not be easy to determine. There is an acute shortage of nephrologists when compared with the growing number of end-stage renal disease patients in our country and in most developing countries. Hence, blindly following the guidelines of developed countries for the early referral of all these patients to nephrologists may not be feasible. In view of this situation, more studies are necessary to determine whether the predialysis care given by primary physicians can be improved by giving additional training to them so that the overall morbidity and mortality of these patients can be improved without overburdening the already overwhelmed nephrology services.
